# The Ferroptosis–Mitochondrial Axis in Depression: Unraveling the Feedforward Loop of Oxidative Stress, Metabolic Homeostasis Dysregulation, and Neuroinflammation

**DOI:** 10.3390/antiox14050613

**Published:** 2025-05-20

**Authors:** Xu Liu, Qiang Luo, Yulong Zhao, Peng Ren, Yu Jin, Junjie Zhou

**Affiliations:** 1 School of Rehabilitation Medicine, Gannan Medical University, Ganzhou 341000, China; liuxu@gmu.edu.cn (X.L.); luoqiang@gmu.edu.cn (Q.L.); zhaoyulong@gmu.edu.cn (Y.Z.); renpeng1@gmu.cn (P.R.); jinyu2@gmu.cn (Y.J.); 2 Key Laboratory of Prevention and Treatment of Cardiovascular and Cerebrovascular Diseases of Ministry of Education, Gannan Medical University, Ganzhou 341000, China; 3 Ganzhou Key Laboratory of Rehabilitation Medicine, Ganzhou 341000, China

**Keywords:** iron dysregulation, ferroptosis, depression, mitochondrial dysfunction, reactive oxygen species, lipid metabolism disorders, disorder of energy metabolism

## Abstract

Emerging evidence links ferroptosis–mitochondrial dysregulation to depression pathogenesis through an oxidative stress–energy deficit–neuroinflammation cycle driven by iron overload. This study demonstrates that iron accumulation initiates ferroptosis via Fenton reaction-mediated lipid peroxidation, compromising neuronal membrane integrity and disabling the GPx4 antioxidant system. Concurrent mitochondrial complex I/IV dysfunction impairs ATP synthesis, creating an AMPK/mTOR signaling imbalance and calcium dyshomeostasis that synergistically impair synaptic plasticity. Bidirectional crosstalk emerges: lipid peroxidation derivatives oxidize mitochondrial cardiolipin, while mitochondrial ROS overproduction activates ACSL4 to amplify ferroptotic susceptibility, forming a self-reinforcing neurodegenerative loop. Prefrontal–hippocampal metabolomics reveal paradoxical metabolic reprogramming with glycolytic compensation suppressing mitochondrial biogenesis (via PGC-1α/TFAM downregulation), trapping neurons in bioenergetic crisis. Clinical data further show that microglial M1 polarization through cGAS-STING activation sustains neuroinflammation via IL-6/TNF-α release. We propose a “ferroptosis–mitochondrial fragmentation–metabolic maladaptation” triad as mechanistic subtyping criteria for depression. Preclinical validation shows that combinatorial therapy (iron chelators + SIRT3 agonists) rescues neuronal viability by restoring mitochondrial integrity and energy flux. This work shifts therapeutic paradigms from monoaminergic targets toward multimodal strategies addressing iron homeostasis, organelle dynamics, and metabolic vulnerability—a framework with significant implications for developing neuroprotective antidepressants.

## 1. Introduction

Depression is a psychiatric disorder manifesting persistent anhedonia (core symptom), cognitive impairment (e.g., executive dysfunction), and psychomotor retardation, with DSM-5 requiring symptom persistence ≥ 2 weeks [1,2]. WHO epidemiological surveillance reveals that major depressive disorder (MDD) currently affects 350 million individuals globally, with its severe manifestations directly contributing to approximately +800,000 annual suicide fatalities, representing 14.3% of all suicide-related mortality [3,4,5]. The multifactorial pathogenesis of depression encompasses (1) monoaminergic neurotransmission deficits, (2) hypothalamic–pituitary–adrenal (HPA) axis dysregulation, (3) cytokine-mediated neuroinflammation (particularly TNF-α signaling), (4) nitric oxide/cyclic GMP pathway aberrations, (5) mitochondrial autophagy–apoptosis interplay, and (6) gene–environment interactions modifying neural plasticity [6,7,8,9]. Emerging evidence implicates dysregulated iron homeostasis, particularly within limbic–cortical circuits vulnerable to ferroptosis, as a pivotal pathogenic determinant in depression through iron-mediated oxidative cascades that disrupt neuronal redox homeostasis [2,4,5,10,11,12].

Ferroptosis is a new form of cell death proposed in 2012 [13]. Ferroptosis arises from dysregulated iron homeostasis driving iron overload, promoting lethal lipid peroxides that compromise GPX4 antioxidant defense while concurrently modulating DHODH, GCH1/BH4/DHFR, and AMPK signaling—key regulators of redox equilibrium collapse preceding iron-dependent membrane disintegration [14,15,16,17,18,19,20,21]. This process not only plays an important role in neurodegenerative diseases but is also closely related to the pathogenesis of depression [22,23]. Cerebral iron disproportionality instigates ROS-mediated oxidative insult and neuroinflammatory cascades via concerted activation of NF-κB/JNK/p38 MAPK axes, with resultant neuronal integrity loss constituting core pathophysiological signatures of depressive disorders [24,25,26,27,28].

Recent years have witnessed burgeoning interest in the tripartite nexus of ferroptosis, bioenergetic derangement, and mitochondrial integrity loss in depression pathogenesis. Ferroptotic progression precipitates mitochondrial cristae disorganization and complex I/II inactivation, thereby crippling Krebs cycle flux (via aconitase suppression), impairing GLUT3-mediated glucose utilization, and instigating catastrophic ATP depletion—events converging on neuronal bioenergetic failure [29]. Moreover, the brain is a highly metabolically active organ, and its normal energy metabolism is essential for the normal functioning of the nervous system [30]. Emerging evidence suggests depression involves bioenergetic homeostasis disruption, particularly prefrontal–limbic hypometabolic states characterized by impaired glycolytic flux and compromised mitochondrial oxidative capacity. This energy crisis potentiates dendritic spine loss and neuroplasticity deficits through redox imbalance-mediated synaptic pruning, creating a self-amplifying loop that accelerates disease progression [31]. Wang and Ahola et al. showed that iron ion overload interferes with the mitochondrial oxidative phosphorylation process by promoting reactive oxygen species (ROS) generation and ultimately leads to a decrease in ATP synthesis [32,33]. Iron overload instigates mitochondrial membrane permeabilization through Fenton reaction-derived hydroxyl radicals that induce lipid peroxidation cascades. This redox imbalance concurrently triggers p53-mediated Bax mitochondrial translocation (apoptotic pathway) and GPX4 inactivation-driven phospholipid hydroperoxide accumulation (ferroptotic pathway), establishing competing cell death modalities [34,35]. Ferrous ions disrupt Keap1-Nrf2-ARE axis function by promoting Nrf2 ubiquitination, thereby compromising phase II detoxification enzyme induction—a critical redox buffering mechanism whose failure perpetuates a self-amplifying oxidative insult [1,24]. Mitochondria, serving as primary bioenergetic hubs, orchestrate interpathway crosstalk between ferroptosis and apoptosis through the iron–redox nexus. In iron-overloaded states, Fe^2+^-driven Fenton reactions generate supraphysiological ROS that (a) activate ASK1-p38/JNK signaling via thioredoxin-1 oxidation and (b) precipitate mitochondrial outer membrane permeabilization (MOMP) through VDAC oligomerization, enabling cytochrome c efflux. These coordinated events culminate in the dual activation of (a) the caspase-9/3 cascade via apoptosome assembly (apoptotic execution) and (b) GPX4 functional ablation through selenocysteine oxidation (ferroptotic initiation) [36,37]. Oxidative stress induces mitochondrial membrane depolarization through cardiolipin peroxidation, coupled with disrupted mitochondrial Ca^2+^ efflux via MCU/RyR dysregulation. This dual insult activates BAX oligomerization while suppressing BCL-2, triggering CASP3-mediated apoptotic execution and impairing oxidative phosphorylation through complex V dysfunction—bioenergetic failures that manifest as synaptodendritic atrophy in mood-regulating circuits, phenocopying core depressive symptomatology [38,39]. In their findings, Stockwell and Uzungil suggest that the complex interplay of ferroptosis, mitochondrial dysfunction, and dysregulated energy metabolism may collectively drive the onset and progression of depression [40,41]. The tripartite nexus among these components establishes novel conceptual frameworks for elucidating the pathogenesis of depression and advancing therapeutic strategies. This review systematically examines their intricate interplay and underlying mechanisms in depression pathophysiology, critically evaluates methodological limitations in current research paradigms, and proposes innovative directions for both fundamental research and therapeutic development.

## 2. The Relationship Between Ferroptosis and Depression

### 2.1. Ferroptosis Revealed: Lipid Peroxidation, Gpx4 Dysfunction and Neuronal Vulnerability in the Context of Neurodegeneration

**Figure 1:**
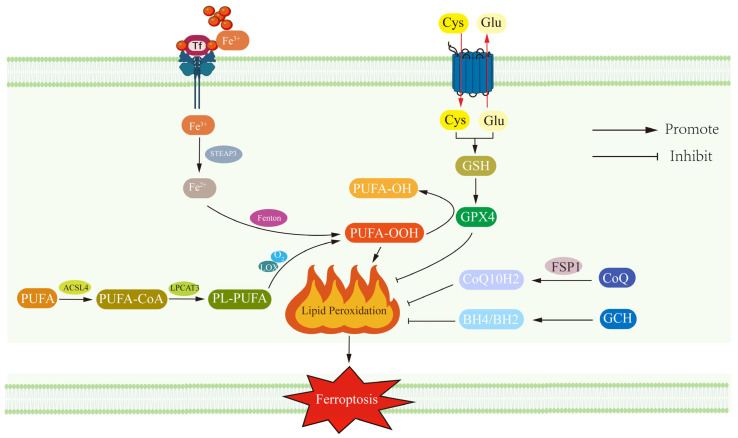
Molecular mechanisms of ferroptosis and key regulatory pathways. This figure systematically elucidates the core driving mechanism of ferroptosis and its inhibitory regulatory network: iron overload catalyzes the lipid peroxidation process of polyunsaturated fatty acids (PUFAs) via the Fenton reaction, in which ACSL4/LPCAT3-mediated PUFA-CoA esterification and phospholipid membrane incorporation are critical for initiation (**left**). System Xc—the GSH-GPX4 antioxidant axis maintains oxidative homeostasis by reducing lipid peroxides (**right**). Important inhibitory pathways include the FSP1/CoQ10H_2_ axis to neutralize lipid free radicals via the ubiquinone regeneration system and the BH4 metabolic pathway to synergistically regulate oxidative stress. The nodes labeled in the figure (e.g., GPX4, FSP1, etc.) are potential therapeutic targets, and their network of interactions provides a molecular basis for ferroptosis-related disease intervention.

Ferroptosis is iron-dependent programmed cell death that is distinct from classical apoptosis, necrosis, and autophagy [42]. Scientists observed a cell death phenomenon with iron-dependent characteristics as early as the 1950s [43,44,45,46,47], It was not until the concept of “ferroptosis” was first introduced by Scott J. Dixon et al. in 2012 that its unique biology was revealed [13]. The mechanism is illustrated in Figure 1. The central pathogenic mechanism of ferroptosis involves the depletion of glutathione (GSH) pools and subsequent enzymatic dysfunction of glutathione peroxidase 4 (GPX4). This critical failure in the redox homeostasis system disrupts GPX4-mediated detoxification of lipid peroxides, culminating in their excessive accumulation within cellular membranes and subsequent initiation of the ferroptotic cascade through peroxidation-driven membrane destabilization [14,16]. Another feature of ferroptosis is the accumulation of ferrous iron (Fe^2+^) ions, which promote lipid peroxidation by catalyzing the generation of free radicals, and ultimately the generation of large amounts of ROS, causing cell membrane rupture and cell death [48,49].

The process of ferroptosis begins with an imbalance between iron import and reduction. Fe^3+^ enters the cell via iron transport proteins and is converted to Fe^2+^ with the aid of STEAP3 reductase [50]. Promoter methylation of FPN1 may inhibit iron efflux and lead to neuronal iron accumulation [51]. Excess intracellular Fe^2+^ generates ROS via the Fenton reaction, which disrupts and accelerates intracellular polyunsaturated fatty acid (PUFA) oxidation [50,52]. In particular, ACSL4 and LPCAT3 significantly elevated the oxidative susceptibility of membrane lipids when combining PUFA with phosphatidylethanolamine to form PUFA-PE (arachidonic acid (AA-PE) with adrenoic acid (AdA-PE)) [27,53]. The catalytic role of lipoxygenase (LOX) puts PUFA-PE at the heart of the oxidation reaction, which further exacerbates ferroptosis [54]. GPX4 is the only enzyme capable of scavenging lipid peroxides in this process, but its activity is dependent on GSH, and once GSH is depleted or GPX4 is impaired, it leads to ferroptosis as lipid peroxides are not scavenged in a timely manner [55,56]. Notably, calcium-independent phospholipase A2 beta (PLA2G6) has emerged as another crucial enzyme in mitigating lipid peroxidation through selective hydrolysis of peroxidized phospholipids, particularly phosphatidylethanolamine (PE) species such as Hp-PE [57,58]. PLA2G6-mediated cleavage of oxidized phospholipids generates lyso-PE and free oxidized fatty acids, thereby interrupting lipid peroxidation cascades. Deficiency in PLA2G6 exacerbates mitochondrial lipid peroxide accumulation and compromises mitochondrial membrane integrity, rendering neurons vulnerable to ferroptotic injury [58]. Beyond the canonical GPX4 pathway, deficiency in ferroptosis suppressor protein 1 (FSP1) emerges as a parallel antioxidant system through its NAD(P)H-dependent reduction of ubiquinone (CoQ10). Suppression of FSP1-mediated redox cycling synergistically compromises cellular defense mechanisms, predisposing neurons to ferroptotic vulnerability by permitting uncontrolled propagation of lipid peroxidation cascades [59,60].

Ferroptosis is particularly important in the nervous system. Iron is essential for normal brain function, but excess iron can trigger oxidative stress and damage neurons [61]. Iron overload demonstrates significant pathological association with neurodegenerative diseases, notably Alzheimer’s disease and Parkinson’s disease, through mechanisms involving redox imbalance and neuronal oxidative damage [22,34,62,63]. Cerebral iron deposition and ferroptotic activation exacerbate neuronal injury through Fenton chemistry-amplified lipid peroxidation cascades and self-propagating ROS overproduction [64]. In conclusion, ferroptosis exhibits multifactorial pathogenesis stemming from the pathophysiological crosstalk between dysregulated iron homeostasis and aberrant lipid peroxidation cascades. Elucidating this redox biology axis extends beyond mechanistic understanding of neurodegenerative pathologies to encompass the identification of druggable targets, thereby bridging fundamental discoveries with therapeutic innovation for developing disease-modifying interventions.

### 2.2. Iron Overload in Depression: A Double-Edged Sword Linking Neurotransmitter Synthesis and Ferroptosis

Iron homeostatic dysregulation constitutes a pathogenic nexus in depression, functioning dually as a ferroptosis initiator and a neuroendocrine disruptor. As an essential cofactor for rate-limiting enzymes (e.g., tyrosine hydroxylase in dopamine synthesis and tryptophan hydroxylase in 5-HT biosynthesis), iron imbalance directly impairs monoaminergic neurotransmission. This metal-mediated disruption of neurotrophic signaling establishes a self-perpetuating pathogenic cycle wherein neurotransmitter deficiencies exacerbate iron mismetabolism, thereby amplifying both oxidative stress and affective disorder progression [65]. Chronic inflammatory states induce hepcidin-mediated suppression of ferroportin (FPN1) expression, culminating in compartmentalized iron mismetabolism characterized by cellular iron retention and paradoxical systemic hypoferremia. Substantiating this dual pathology, Wang et al. (2023) revealed a paradoxical disequilibrium in depressive disorders where serum iron profiles concurrently exhibit features of tissue-level iron overload and circulatory iron deficiency, reflecting disrupted systemic iron redistribution mechanisms [66]. Iron deficiency disrupts dopamine synthesis via tyrosine hydroxylase inhibition, while iron overload triggers Fenton-mediated oxidative damage. Their interplay creates a self-perpetuating cycle of monoaminergic dysfunction and neuronal redox stress [66,67].

At the molecular nexus of iron–neurotransmitter crosstalk, iron serves as a critical enzymatic cofactor in monoaminergic neurotransmission through allosteric modulation of rate-limiting biosynthetic enzymes. Specifically, ferrous ions (Fe^2+^) regulate the catalytic competence of tyrosine hydroxylase (TH; EC 1.14.16.2) and tryptophan hydroxylase 2 (TPH2; EC 1.14.16.4), governing dopamine and serotonin (5-HT) biosynthesis, respectively. Paradoxically, 5-HT exhibits antioxidant neuroprotection via TrkB receptor-dependent potentiation of glutathione (GSH) synthesis. Conversely, deficits in TrkB signaling impair glutamate-mediated xCT activation (system Xc-), thereby suppressing cystine uptake and exacerbating ferroptotic vulnerability through GPX4 functional depletion [68,69]. Iron deficiency can affect the activity of these enzymes, and restricted functioning of these enzymes can lead to decreased synthesis of neurotransmitters, worsening depressive symptoms [68]. Iron is also involved in a variety of responses related to neuroprotection. For example, iron participates in intracellular redox reactions by regulating the activity of iron–sulfur proteins, promoting neuronal resistance to oxidative stress [70]. Iron also interacts with molecules such as brain-derived neurotrophic factor (BDNF) to jointly maintain neuroplasticity and protect normal functions [71].

Iron accumulation demonstrates a significant pathological correlation with oxidative lesion propagation. Redox-active iron pools, particularly labile Fe^2+^ ions, drive Fenton chemistry-mediated generation of hydroxyl radicals (^•^OH) through the redox cycling between Fe^2+^ and H_2_O_2_:

Fe^2+^ + H_2_O_2_ → Fe^3+^ + ^•^OH + OH^−^.

These hyperreactive oxidants, including the diffusion-limited ^•^OH (half-life ~1 ns), induce peroxidative chain reactions in polyunsaturated fatty acid (PUFA)-rich lipid bilayers while concurrently mediating oxidative DNA base modifications (e.g., 8-oxoguanine) and protein carbonyl adduct formation [5,24,72,73,74]. The brain’s metabolic vulnerability exacerbates iron-overload-induced oxidative damage. Specifically, Fenton reaction-mediated lipid peroxidation in mood-regulatory centers—particularly the prefrontal cortex (executive function) and hippocampus (neurogenesis)—disrupts synaptic plasticity through impaired dendritic spine remodeling and monoaminergic circuit dysfunction, thereby accelerating the neurodegenerative cascade in depressive pathophysiology [75,76,77]. Excess iron affects the structure and function of nerve cells at the molecular level by participating in the Fenton reaction that catalyzes the formation of oxidative free radicals and leads to lipid peroxidation, which damages the phospholipids of the cell membrane [78].

Ferroptosis as a new iron-dependent cell death mechanism has been recognized as one of the important pathways in the development of depression [10,11,12]. Ferroptosis executes cellular demise through peroxidative disintegration of polyunsaturated fatty acid (PUFA)-enriched membranes—a process mechanistically driven by ACSL4/LPCAT3-mediated PUFA enrichment and dysfunctional glutathione peroxidase 4 (GPX4)-mediated redox homeostasis. This ferroptotic cascade synergistically amplifies oxidative injury via LOX/ALOX15 enzymatic propagation of lipid peroxides while concurrently activating neuroinflammatory responses through HMGB1/TLR4-mediated microglial pyroptosis, thereby establishing a self-amplifying pathogenic loop linking membrane failure, redox collapse, and neuroimmune dysregulation [78,79]. Ferroptosis engages in bidirectional crosstalk with neuroinflammatory pathways, wherein pro-inflammatory cytokines (e.g., TNF-α, IL-6) establish feedforward loops through redox-sensitive signaling. TNF-α activates the canonical NF-κB signaling axis to upregulate iron import machinery (divalent metal transporter 1 [DMT1] and transferrin receptor 1 [TfR1]), while transcriptionally repressing ferroportin (FPN1)-mediated iron export. This cytokine-driven dysregulation of neuronal iron homeostasis results in pathological iron retention, synergistically amplifying lipid peroxidation cascades and depressive-like phenotypes through neuroinflammatory–oxidative comorbidity pathways [12,80].

Iron dyshomeostasis demonstrates pathogenic convergence between depression and neurodegenerative disorders, with clinical studies by Onukwufor et al. (2022), Ma et al. (2022), and Tarnacka et al. (2021) revealing comorbid neurodegenerative pathologies in depressed populations. Mechanistically divergent yet etiologically parallel, Alzheimer’s disease ferroptosis manifests β-amyloid (Aβ)-mediated peroxidation of phospholipid membranes rich in docosahexaenoic acid (DHA), while depressive disorder ferroptosis originates from monoaminergic neurotransmission collapse via iron-dependent inhibition of tryptophan/tryptophan hydroxylase systems. Crucially, both conditions share terminal neurodegeneration pathways through iron-overaccumulation-induced NLRP3 inflammasome activation and NADPH oxidase-2 (NOX2)-derived superoxide overproduction, ultimately converging on synaptic elimination via oxidative modification of BDNF-TrkB signaling cascades [22,34,81,82].

## 3. Mitochondrial–Energy Metabolism Dysregulation in Depression

### 3.1. Mitochondria at the Central Hub: Bioenergetic Failure and ROS-Driven Synaptic Dysfunction in the Pathogenesis of Depression

The development of depression is closely related to the dysregulation of energy metabolism. Although the brain accounts for only 2% of the body’s mass, its energy consumption accounts for 20% of the body’s total oxygen consumption [83,84]. The brain is thus a highly energy-dependent organ and its energy needs must be finely regulated. Neuronal bioenergetics predominantly rely on glucose as the primary metabolic substrate, with the CNS maintaining obligatory glycolytic dependence even under catabolic stress. Alternative fuel utilization (e.g., astrocyte–neuron lactate shuttle, ketolytic pathways) remains governed by stringent regulatory mechanisms that prioritize redox homeostasis and synaptic transmission fidelity over energy yield optimization, thereby safeguarding oxidative metabolism-coupled neurocognitive integrity [85,86,87]. This high dependence on energy supply causes neuronal function to be quickly impaired when the brain encounters metabolic disorders, especially when glucose supply is limited, leading to severe impairments in affective and cognitive functioning [88,89].

From inside the cell, ATP is a key energy carrier for maintaining neuronal function [90]. ATP synthesis is mainly dependent on the oxidative phosphorylation (OXPHOS) pathway in mitochondria [90,91]. NADH and FADH_2_ release energy in this process through a series of electron transfer chain reactions that consume oxygen and ultimately produce ATP. Mitochondria are not only responsible for ATP synthesis, but are also involved in the regulation of lipid metabolism, free radical scavenging, and apoptosis [38,39,92]. Mitochondrial bioenergetic competence in neurons is indispensable for sustaining action potential propagation and Ca^2+^-dependent exocytotic neurotransmitter release. The uninterrupted ATP provision through oxidative phosphorylation (OXPHOS) constitutes a non-negotiable metabolic requisite for maintaining neuronal excitability thresholds and preventing activity-dependent redox collapse in high-firing neural networks [30,31]. Inadequate ATP synthesis in impaired mitochondrial function will directly affect neuronal activity, leading to disruption of the metabolic homeostasis of the cell and potentially triggering ferroptosis [93,94]. Thus, depressed patients often exhibit impairment of mitochondrial function and imbalances in energy metabolism, a state that both affects basic physiological cellular function and is closely linked to the pathogenesis of the disease.

Recent studies have also shown that imbalances in mitochondrial dynamics also play a key role in disturbed energy metabolism in depression. Overactivation of the mitochondrial-splitting protein Drp1 leads to mitochondrial fragmentation, inhibits OXPHOS efficiency and reduces ATP synthesis [95,96,97]. Clinically derived metabolomic signatures reveal marked lactate depletion within hippocampal subfields of major depressive disorder (MDD) cohorts, indicating astrocytic glycolytic insufficiency disrupts the astrocyte–neuron lactate shuttle (ANLS) axis. This glial metabolic deficiency deprives neurons of lactate-derived pyruvate for mitochondrial oxidative phosphorylation while concurrently impairing lactate-mediated epigenetic regulation of antioxidant genes (e.g., Nrf2), thereby synergistically exacerbating neuronal redox stress and bioenergetic collapse in mood-regulatory circuits [98,99]. Emerging evidence highlights mitochondria as central regulators of cellular iron homeostasis. Dysfunctional mitochondrial networks impair Fe-S cluster biogenesis and heme synthesis—processes essential for OXPHOS integrity. This disrupts iron-buffering capacity, leading to pathological iron accumulation that amplifies lipid peroxidation cascades and ferroptosis susceptibility [100,101]. In addition, reduced mitochondrial complex I (NADH dehydrogenase) and IV (cytochrome C oxidase) activities, leading to decreased NADH/FADH_2_ production, can be verified by decreased [18F]FDG uptake in PET imaging of the brain [102].

### 3.2. Metabolic Crisis Meets Lipid Peroxidation: How Glucose and Fatty Acid Dysregulation Promotes Ferroptosis in Depression

**Figure 2:**
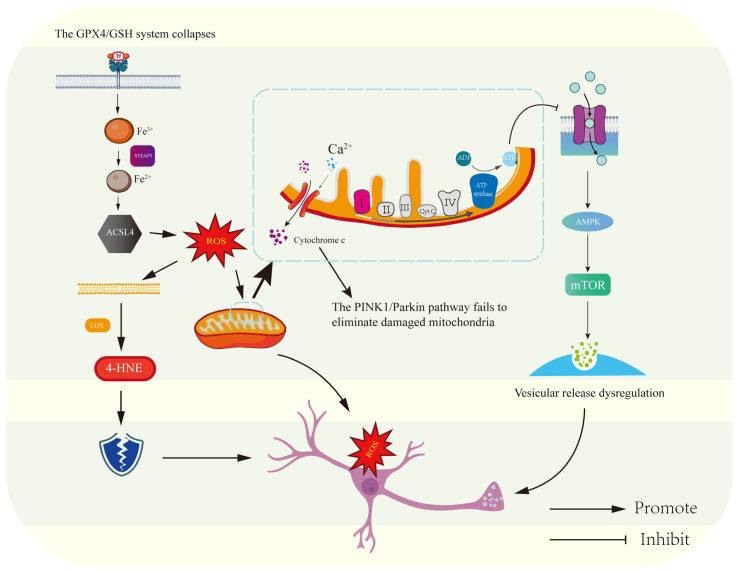
Interactive mechanisms of ferroptosis, mitochondrial dysfunction and metabolic dysregulation in depression. This figure integrates three core mechanisms in the pathological process of depression: (1) ferroptosis: iron accumulation in brain regions triggers the Fenton reaction, leading to lipid peroxidation of polyunsaturated fatty acids (PUFAs), and oxidative imbalance is exacerbated by reduced activity of GPX4; (2) mitochondrial dysfunction: TFAM/PGC-1α-mediated mitochondrial biogenesis is impaired, and abnormalities in the respiratory chain complex (I/IV) result in reduced ATP reduced synthesis and excessive accumulation of ROS; (3) metabolic dysregulation: reduced glucose uptake in prefrontal/hippocampal regions (GLUT4 downregulation), and dysregulation of mTOR-mediated vesicle release resulting in neurotransmitter disorders. The three form a pathological network through damage to mitochondrial membranes by lipid peroxidation products, a positive feedback loop of ROS–iron accumulation, and a synergistic effect of energy metabolism collapse–oxidative stress.

Depression is a psychiatric disorder with a multifactorial pathogenesis that involves multiple cellular, molecular, and metabolic pathway abnormalities in addition to neurotransmitter imbalances; the core mechanism underlying the pathological progression of depression is illustrated in Figure 2. Abeysekera has shown in his recent studies that disorders of glucose metabolism play a key role in the onset and progression of depression [103]. Glucose is the main source of energy supply for the brain, while neurons are highly dependent on glucose transporter proteins (GLUTs) in the exercise of their normal functions [104,105]. Neurovascular metabolic studies by Koepsell et al. and Bigio et al. demonstrate significant downregulation of cerebral glucose transporters (GLUT1 in blood–brain barrier endothelia; GLUT3 in neuronal membranes) in major depressive disorder. This transporter deficiency impairs astrocytic–endothelial coupling for glucose shuttling, creating a hippocampal–prefrontal hypometabolic state that disrupts ATP-dependent synaptic vesicle cycling and monoamine reuptake machinery—providing a pathophysiological substrate for depression’s core behavioral manifestations including psychomotor retardation and affective flattening [103,105]. On the other hand, insulin, as a metabolic regulator, can regulate glucose uptake and metabolism in cells through the PI3K/Akt signaling pathway [106,107]. However, depression is often accompanied by insulin resistance, which can lead to impairment of this normal pathway and affect glucose metabolism [107]. In addition to interfering with glucose metabolism, insulin resistance may also affect neuronal membrane potential, synaptic plasticity and other neural functions, further exacerbating depression [108]. Dysregulation of the PI3K/Akt signaling axis precipitates neuronal bioenergetic crisis through impaired insulin-mediated glucose transporter trafficking and defective mitophagy, establishing a self-amplifying ROS-mediated neurotoxic cycle that perpetuates synaptic ATP depletion while exacerbating depressive symptomology through oxidative phosphorylation-coupled neuroplasticity failure [108].

Abnormal fatty acid metabolism also plays a key role in the mechanism of depression. Fatty acids serve dual roles as integral structural constituents of neuronal membranes and as dynamic mediators of neuroenergetic homeostasis. Polyunsaturated fatty acids (PUFAs) like docosahexaenoic acid (DHA) not only maintain membrane fluidity for synaptic vesicle cycling but also undergo β-oxidation in neuronal mitochondria to generate ATP-equivalent reducing equivalents (NADH/FADH_2_), while simultaneously functioning as endogenous ligands for nuclear receptors (PPARγ) that coordinate antioxidant defense programs against lipid peroxidation cascades [109]. Under normal conditions fatty acids enter the mitochondria through β-oxidation and generate ATP to provide energy for life activities [109,110]. However, depressed patients often exhibit a decreased ability to oxidize fatty acids. This change not only affects cellular energy supply by reducing ATP synthesis, but also impairs mitochondrial function, which in turn affects neuronal survival and function [111]. Impairment of fatty acid metabolism may also trigger lipid accumulation and activate the inflammatory response of the nervous system to aggravate the pathological process of depression, and the regulators of fatty acid metabolism play an important role in the maintenance of fatty acid metabolism homeostasis [112]. For example, PPAR-α is one of the regulators of fatty acid metabolism, which can inhibit inflammation and improve antioxidant function by regulating the transport and oxidation of fatty acids, and can play a role in protecting neuronal cells [113]. PPAR-α transcriptional dysregulation precipitates a dual neuropathological cascade: compromised fatty acid β-oxidation induces mitochondrial bioenergetic insufficiency through CPT1A-mediated carnitine shuttle impairment, while defective PPAR-α-mediated anti-inflammatory signaling unleashes TLR4/NF-κB-driven neuroinflammation via unopposed arachidonic acid metabolite (prostaglandin E2/leukotriene B4) overproduction. This neuroimmune–metabolic nexus establishes a self-reinforcing loop where lipidomic imbalance and microglial priming synergistically propel depression pathogenesis through oxidative eicosanoid storm and synaptic ATP crisis [114,115].

Ferroptosis in depression is closely linked to abnormalities in lipid metabolism. ACSL4 promotes lipid peroxidation and triggers ferroptosis by catalyzing the esterification of long-chain polyunsaturated fatty acids (PUFAs) to phospholipids [116], whereas mitochondrial ROS further amplify ACSL4-mediated membrane damage by oxidizing mitochondrial cardiolipin [117,118]. Notably, reduced cysteine uptake due to insulin resistance inhibits glutathione (GSH) synthesis and reduces GPX4 activity, thereby disarming the inhibition of lipid peroxidation [119]. Furthermore, dysregulation of PPAR-α synergy with PGC-1α reduces fatty acid oxidase (e.g., CPT1A) expression, exacerbates lipotoxicity, and activates the TLR4/NF-κB inflammatory pathway [120].

The development of depression is also closely related to imbalances in calcium ion homeostasis, oxidative stress, ferroptosis, and disturbances in neurotransmitter metabolism by a variety of pathological mechanisms. Calcium ions are involved in signaling and synaptic plasticity in neuronal cells and maintain normal cellular function. However, intraneuronal calcium overload in depression precipitates neuronal hyperexcitability via CaMKII/calcineurin axis activation, driving excitotoxic cascades through NMDA receptor hypersensitization. This calcium dyshomeostasis triggers mitochondrial bioenergetic failure via mPTP opening and cytochrome c efflux, culminating in BAK/BAX-mediated apoptosome activation and caspase-9-dependent apoptotic execution—a self-amplifying neurotoxic loop linking glutamate excitotoxicity, ATP synthase dysfunction, and oxidative DNA fragmentation [121,122,123]. Calcium ion imbalance is also closely related to oxidative stress. Elevated levels of ROS triggered by oxidative stress not only damage cell membranes, proteins, and DNA, but also promote ferroptosis by regulating iron metabolism [124]. Disorders of tryptophan metabolism and imbalances of glutamate and GABA also play a key role in depression [125]. Serotonin (5-hydroxytryptamine, 5-HT) functions as the pivotal monoaminergic neurotransmitter governing affective homeostasis, with its biosynthesis contingent upon tryptophan hydroxylase (TPH)-mediated conversion of dietary tryptophan to 5-hydroxytryptophan (5-HTP). Deficits in tryptophan bioavailability—whether from malnutrition, indoleamine 2,3-dioxygenase (IDO)-mediated diversion into kynurenine pathways, or TPH2 epigenetic silencing—provoke serotonergic synaptic insufficiency. This manifests as altered glutamatergic/GABAergic tone and monoaminergic receptor hypersensitivity, thereby potentiating corticolimbic circuit excitotoxicity while concomitantly activating neuroinflammatory–astrocytic feedback loops that drive affective disorder pathogenesis through oxidative tryptophan metabolite accumulation [126]. On the other hand, excessive release of glutamate triggers neurotoxicity, while a decrease in GABA weakens the brain’s inhibitory function, and both alterations can lead to symptoms such as low mood and cognitive impairment [125]. These factors mentioned above are intertwined to drive the onset of depression.

### 3.3. Mitochondrial Extinction in Depression: From Membrane Permeability Changes to Apoptosis

Mitochondria play a central role in cellular bioenergetics, orchestrating the oxidative phosphorylation (OXPHOS) process that generates ATP as the universal energy currency for physiological functions [92,111]. NADH and FADH_2_ transfer electrons through the respiratory chain complex in mitochondria, producing water in the presence of oxygen and driving proton transport across the membrane to form an electrochemical gradient, which is used by ATP synthase to synthesize ATP [30]. ATP is not only a direct energy source for cellular metabolism, but also regulates cellular physiological processes. Mitochondria are also involved in the regulation of calcium homeostasis, oxidative stress response and cell death, and the health of mitochondria in the nervous system is critical to neuronal survival and function [39,86]. Calcium channels on the inner mitochondrial membrane (e.g., MCU) regulate calcium ion concentration; impairment of mitochondrial membrane potential or ATP synthesis disrupts calcium homeostasis and triggers a pathological response; high levels of calcium ions activate esterases and ATPases to exacerbate cellular damage [127]. Mitochondria can also release molecules such as cytochrome C to initiate apoptotic programs to regulate cell death [128]. In conclusion, mitochondrial dysfunction can lead to a variety of diseases. In experimental models of depression, stress-induced impairment of TFEB (a master regulator of autophagy) nuclear translocation has been shown to disrupt lysosomal–autophagic flux, thereby significantly aggravating the accumulation of dysfunctional mitochondria [129]. Abnormal mitochondrial calcium unidirectional transporter (MCU) function leads to matrix calcium overload, activation of mPTP opening, and release of cytochrome C [130]. Meanwhile, the structural disorganization of endoplasmic reticulum–mitochondria contact sites (MAMs) compromises intracellular calcium homeostasis, culminating in pathological activation of IP3R-VDAC1 channel-mediated mitochondrial calcium overload [131].

Mitochondrial damage is closely related to impairment of membrane integrity. Hypoxia, oxidative stress, or toxic stimulation can cause changes in membrane permeability and lead to abnormal accumulation of calcium ions and loss of membrane potential, which inhibits ATP synthesis and disrupts the normal supply of cellular energy [132]. Mitochondrial dysfunction facilitates cytochrome c efflux via mitochondrial permeability transition pore (mPTP) channels, a process that has been demonstrated to activate caspase-9 (cysteine–aspartic proteases) and subsequently initiate programmed apoptotic pathways or necrotic cell death through defined pathological cascades [133]. MPTP opening also leads to rupture of the outer membrane, which promotes leakage of macromolecules such as cytochrome C, further exacerbating cell death [133,134]. Selective mitophagy, a quality control mechanism for eliminating dysfunctional mitochondria, is primarily mediated by the PINK1–Parkin signaling axis. This pathway orchestrates mitochondrial clearance through ubiquitin-dependent labeling of damaged organelles, facilitating their targeted sequestration within autophagosomes, thereby preventing cytotoxic cascade initiation through containment of mitochondrial-derived danger signals [135]. Mitochondrial autophagy prevents oxidative stress and cell death by maintaining cellular energy homeostasis, and dysregulated autophagy is a key factor in neurodegenerative diseases such as Alzheimer’s disease and Parkinson’s disease [136,137].

Mitochondria in the nervous system are not only involved in energy metabolism, but also regulate neuronal function through interactions with astrocytes. Ni et al. demonstrated that intercellular mitochondrial transfer occurs through two distinct mechanisms: tunneling nanotube (TNT)-mediated intercellular contacts and microtubule-associated trafficking mechanisms. These transferred mitochondria functionally mediate the restoration of ATP synthesis, stabilization of calcium ion homeostasis, and promote neuroplastic remodeling through energy-dependent cytoskeletal rearrangement [138]. Mitochondrial health is critical for synaptic function and synaptic plasticity in the hippocampus, and mitochondrial dysfunction can lead to synaptic loss and affect cognitive functions [139]. Gowda and Deng et al. also demonstrated that mitochondrial dysfunction in psychiatric disorders such as depression leads to reduced ATP, decreased efficiency of oxidative phosphorylation, and impacts neuronal survival and synaptic plasticity [130,140]. On the other hand, oxidative stress increases the level of ROS, which also damages mitochondrial membranes, DNA, and lipids and accelerates mitochondrial senescence, further affecting neuronal function and exacerbating neurological pathology [5,24,36,73].

### 3.4. Broken Energy Reservoirs: Mitochondrial ROS, Dysregulated Calcium Homeostasis, and the Neuroinflammatory Cascade in Depression

Mitochondria are essential organelles responsible for ATP synthesis, calcium ion regulation, and redox homeostasis [30]. Under the pathology of depression manifested as a decrease in the number of mitochondria in the hippocampal brain region and impaired function of the respiratory chain in the brain, Chen and Song have demonstrated that mitochondrial dysfunction is a key mechanism for both of these alterations [38,39]. These dysfunctions not only reduce the energy supply to neurons, but also exacerbate the accumulation of ROS, creating another vicious cycle [141]. Elevated ROS damage cell membranes, DNA, and proteins through lipid peroxidation, which leads to endoplasmic reticulum stress and calcium overload by reducing the cell’s buffering capacity for calcium ions [5,24]. Calcium dyshomeostasis elicits progressive neuronal injury via dual pathogenic pathways: (1) direct potentiation of excitotoxic damage through sustained membrane depolarization, and (2) metabolic derangement mediated by hyperactivation of calcium-sensitive effector systems including the Na^+^/Ca^2+^ exchanger (NCX) and protein kinase C (PKC) isoforms. These calcium-dependent signaling cascades have been mechanistically linked to neurodegenerative pathophysiology in depression through their capacity to disrupt mitochondrial bioenergetics and promote oxidative stress, thereby establishing a pathological loop that exacerbates neuroplasticity impairment [121].

Disturbances in mitochondrial function (especially in energy supply and calcium regulation) directly affect neuronal survival and function. Mitochondria experiencing membrane potential dissipation and calcium overload undergo structural transition through opening of the mitochondrial permeability transition pore (mPTP). This pathological pore formation increases ionic permeability, enabling cytochrome c matrix-to-cytoplasm translocation. The liberated cytochrome c subsequently activates initiator caspase-9 (cysteine–aspartic proteases) through binding with apoptotic protease-activating factor-1 (Apaf-1), which oligomerizes to assemble the apoptosome complex. This molecular platform then activates executioner caspases-3/7 via proteolytic cleavage, initiating the intrinsic apoptosis cascade that culminates in programmed cellular demise [133,142,143]. On the other hand, with the decline in ATP synthesis, the intracellular calcium ion concentration gradually increases, which in turn activates calcium-dependent enzymes such as phospholipase A2 to promote further loss of membrane potential [144,145]. Calcium ion accumulation exacerbates cellular damage through the Ca^2+^/calmodulin-dependent protein kinase (CaMKII) pathway and the NF-κB-associated inflammatory pathway, in addition to leading to a collapse of the membrane potential [144]. Excess calcium ions also activate zinc ion channels (e.g., TRPM7) and calcium release from the endoplasmic reticulum, triggering endoplasmic reticulum stress and further exacerbating cellular damage [146]. Mitochondrial membrane potential collapse serves as a potent inducer of autophagic activation, wherein core autophagy machinery components (e.g., LC3-II-mediated autophagosome formation and p62/SQSTM1-dependent cargo recognition) coordinate lysosomal proteolytic activity. This self-catabolic process facilitates the selective elimination of damaged cellular constituents through amphisome–lysosome fusion events, thereby preserving metabolic equilibrium via intracellular quality control surveillance systems [147,148]. However, overactivation of autophagy or defective lysosomal function may result in failure of autophagosomes to fuse with lysosomes, and incompletely degraded substances in the cytoplasm may exacerbate cellular stress and promote apoptosis or necrosis [147]. Depressed patients often exhibit neuronal loss clinically, potentially suggesting that mitochondrial dysfunction plays a key role in this process [39].

Mitochondria also influence synaptic plasticity and neural network function. Mitochondria provide energy through oxidative phosphorylation to support neurotransmitter synthesis and release influencing synaptic transmission efficiency [149]. ATP serves dual critical roles in neuronal physiology: (1) as the bioenergetic substrate for neurotransmitter biosynthesis and exocytotic release, and (2) as a molecular regulator of presynaptic vesicular trafficking and postsynaptic receptor modification cascades. In major depressive disorder (MDD) patients, mitochondrial dysfunction results in bioenergetic deficits characterized by insufficient ATP generation. This metabolic insufficiency compromises interneuronal communication dynamics through three principal pathways: (1) impairment of synaptic vesicle cycling fidelity, (2) dysregulation of membrane fusion machinery, and (3) desensitization of neurotransmitter receptors. Collectively, these ATP-dependent failures disrupt synaptic architecture and plasticity maintenance mechanisms, particularly vesicular docking/release kinetics in presynaptic membranes and ligand-gated ion channel responsiveness in postsynaptic densities within prefrontal–limbic circuits. These neuroenergetic disturbances are mechanistically linked to the pathophysiological substrate underlying depressive symptomatology [39,150,151]. Mitochondria critically orchestrate synaptic plasticity mechanisms through precise modulation of calcium ion dynamics, particularly governing the molecular cascades underlying long-term potentiation (LTP) and long-term depression (LTD). This spatiotemporal control of calcium signaling enables activity-dependent synaptic remodeling in emotion-processing neural circuits, with mitochondrial functional integrity in prefrontal–amygdala pathways being indispensable for maintaining adaptive neuroplastic responses to emotional stimuli [152].

**Figure 3:**
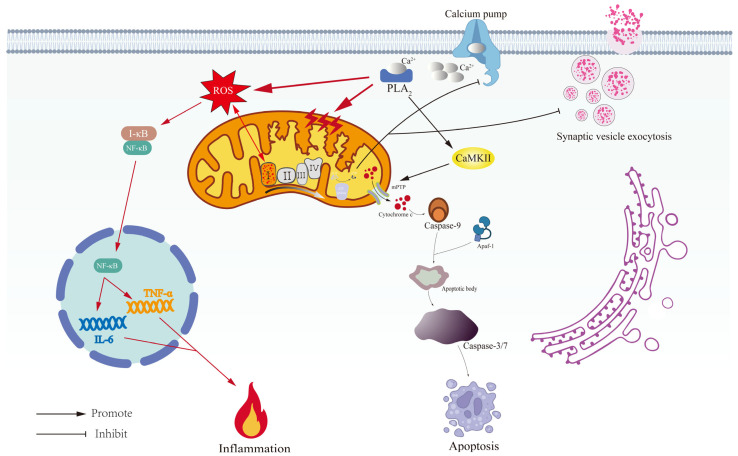
Mitochondrial dysfunction and metabolic dysregulation in depression. This schematic diagram systematically illustrates the pathological mechanism of the mitochondrial–metabolic axis in depression. (i) Mitochondrial structural abnormalities (cristae breaks/mitochondrial swelling) lead to dysfunction of the electron transport chain (complex I–IV), which triggers a decrease in ATP synthesis and a burst of ROS; (ii) ROS upregulate pro-inflammatory factors, such as TNF-α/IL-6, through the activation of NF-κB signaling, resulting in the formation of the neuroinflammation–oxidative stress vicious cycle; (iii) dysregulation of the calcium pump causes intracellular Ca^2+^ overload, which affects neurotransmitter metabolism through the CaMKII signaling cascade; (iv) PLA_2_ is calcium-activated to release peroxidative polyunsaturated fatty acids (PUFAs), which amplify mitochondrial lipid peroxidation (orange ROS burst) and promoting NF-κB-mediated neuroinflammation (red star), thereby propagating oxidative damage and synaptic dysfunction in depression pathogenesis; (v) mitochondrial permeability transition leads to caspase overload, which affects neurotransmitter metabolism through the CaMKII signaling cascade; and (vi) mitochondrial permeability transition leads to the activation of caspase-9/3 apoptotic vesicles, which ultimately triggers programmed cell death. Together, the multidimensional mechanisms form the molecular basis of metabolic destabilization in depression.

The interaction between mitochondrial dysfunction and neuroinflammation also provides new insights into the pathogenesis of depression. Damaged mitochondria trigger a pathological cascade characterized by (1) elevated ROS generation, (2) ROS-mediated oxidative modification of IκB proteins leading to NF-κB activation and subsequent upregulation of pro-inflammatory gene transcription, and (3) sustained release of key inflammatory mediators including TNF-α, IL-6, and NO. These interconnected mechanisms establish a self-perpetuating triad of energy depletion, oxidative stress, and inflammatory activation that progressively impairs cellular homeostasis [48,153,154]. The prefrontal cortex (PFC) exhibits a metabolic reprogramming phenomenon of enhanced glycolysis and OXPHOS inhibition in depression, resulting in decreased expression of synaptic plasticity-related proteins (e.g., PSD-95, Synapsin-1) [155,156]. Liberated mitochondrial DNA (mtDNA) activates the cGAS-STING signaling axis, thereby potentiating type I interferon responses through IRF3 phosphorylation. Concurrently, these escaped mitochondrial genomes synergistically promote the secretion of pro-inflammatory cytokines including IL-6 and TNF-α via TLR9-dependent and -independent mechanisms, establishing an amplification loop between innate immune activation and inflammatory mediator production [143,157,158]. Microglia M1 polarization switches to glycolytic metabolism and further amplifies neuroinflammation through HIF-1α-dependent pathways [159]. This not only impairs neuronal function but may also enhance the inflammatory response of the nervous system by altering glial cell function, which further exacerbates the clinical manifestations of depression; the predominantly affected pathways are illustrated in Figure 3.

## 4. The Iron–Mitochondrial–Metabolic Triad: A Self-Enhancing Network Driving Neuronal Dysfunction in Depression

Ferroptosis, mitochondrial damage, and energy metabolism imbalances are intertwined in depression to form a complex molecular network that drives neuronal dysfunction and mood disorders [3,107]. These mechanisms interact directly at the molecular level to affect cell survival and synaptic function, ultimately interfering with the brain’s ability to regulate emotions. Liu, Ward and Zhang showed that ferroptosis triggers mitochondrial damage and energy metabolism imbalance through enhanced oxidative stress [22,160,161]. As iron accumulation and oxidative damage increase, the onset and progression of depression become more complex [10,11]. Studying the interactions of these mechanisms can help to reveal the pathophysiological process of depression and provide new ideas for future clinical treatment.

Iron overload is one of the central factors in this process. Although iron is an essential element in cells, in excess, it generates ROS through the Fenton reaction, a process that is one of the key pathogenic mechanisms of depression [2,5]. ROS cause neurotransmitter synthesis disorders by oxidizing neurotransmitter synthases (e.g., tyrosine hydroxylase, tryptophan hydroxylase) [162]. Specifically, oxidation of tyrosine hydroxylase (TH) by ROS reduces its catalytic activity through sulfhydryl group modification and iron–sulfur cluster damage, thereby limiting dopamine and norepinephrine synthesis. Similarly, tryptophan hydroxylase 2 (TPH2), the rate-limiting enzyme for serotonin production, is susceptible to ROS-induced structural destabilization due to iron-dependent hydroxyl radical attack on its tetrahydrobiopterin cofactor. These enzyme dysfunctions reflect broader metabolic dysregulation beyond neurotransmitter alterations, as mitochondrial energy metabolism (e.g., ATP depletion) and lipid peroxidation cascades further impair monoamine synthesis pathways [163]. ROS also directly damage cell membranes to trigger ferroptosis [164]. Lipid peroxidation leads to ferroptosis, disrupts the integrity of cell membranes, and triggers an inflammatory response [52]. More importantly, iron overload activates the PI3K/Akt pathway to drive lipid peroxidation and 4-HNE formation, directly compromising membrane integrity through phospholipid oxidation and cholesterol bilayer disorganization [165]. Iron overload also increases ROS production through activation of the NADPH oxidase complex while further exacerbating oxidative stress and ferroptosis [161]. In addition to exacerbating localized neuroinflammation, the released inflammatory factors (e.g., TNF-α and IL-6) amplify the injury by negatively affecting peripheral neuronal survival through the NF-κB pathway [12,36]. Damage to mitochondria is critical in the processes described above, especially in the context of iron overload, which threatens the stability and function of the mitochondrial membrane [40].

Mitochondrial damage is an important link in this large network of mechanisms. This is because mitochondria are not only the energy factories of the cell but also play a key role in regulating redox state and calcium homeostasis [39]. Iron overload and oxidative stress directly disrupt mitochondrial membranes to increase membrane permeability, thus affecting the efficiency of ATP synthesis [38]. ROS inhibit the oxidative phosphorylation process by oxidatively modifying mitochondrial complex I, further reducing ATP production. Oxidative damage also induces autophagy by increasing mitochondrial membrane permeability, which further impairs mitochondrial function [147,148]. Aberrant activation of the mitochondrial-splitting protein Drp1 promotes mitochondrial fragmentation, leading to electron transport chain (ETC) dysfunction and reduced ATP production, whereas deletion of the fusion protein Mfn2 exacerbates mitochondrial DNA (mtDNA) damage and induces neuroinflammation by activating the cGAS-STING pathway [95,97,166]. In addition, defective mitochondrial autophagy leads to the accumulation of damaged mitochondria, which activate NLRP3 inflammatory vesicles by releasing mtDNA, creating a vicious cycle of inflammation–oxidative stress [167]. For neurons, mitochondrial dysfunction not only reduces ATP production, but also severely affects neuronal electrical signaling and synaptic function, inhibits microglia activity, and affects the release of glutamate from presynaptic neurons, which destabilizes neural networks even more and exacerbates clinical symptoms of depression [131,150,168].

Imbalances in energy metabolism are catalytic factors in this complex network of mechanisms. Impaired mitochondrial function leads to reduced ATP synthesis, which in turn affects neurotransmitter synthesis and release [30,31]. Mitochondrial damage forces neurons to enhance glycolysis in dependence on the Warburg effect, leading to lactate accumulation and a decrease in the NAD⁺/NADH ratio, which inhibits mitochondrial biosynthesis driven by the SIRT1/PGC-1α pathway [169,170]. This metabolic reprogramming further reduces neuronal antioxidant capacity and exacerbates ferroptosis sensitivity. Carrard et al. showed that clinically depressed patients often exhibit abnormal energy metabolism in the cerebral cortex and limbic system, and that energy deficits have a direct impact on neuronal activity, leading to decreased synaptic transmission [171]. Mitochondrial dysfunction also inhibits the activation of the AMPK pathway, which reduces the cell’s ability to adapt to energy stress and further impairs neuronal metabolism and viability [172]. AMPK also inhibits the mTORC1 pathway by activating the TSC1/2 complex, which regulates the cellular energetic stress response [172]. Although AMPK activation enhances energy stress adaptation in the short term, chronic activation leads to impaired synaptic plasticity by reducing protein synthesis through inhibition of mTORC1, whereas inactivation of mTORC2 impairs AKT signaling and exacerbates neuronal apoptosis [173]. An experimental study by Dong et al. also confirmed that ATP levels in brain tissues of depressed rat populations were significantly lower than those of normal rat populations, a finding that further validates the relevance of ferroptosis and mitochondrial damage in the clinic [174,175].

It should be noted that the relationship between ferroptosis, ROS accumulation, and energy metabolism imbalance is not a unidirectional causal relationship, but a mutually reinforcing feedback loop. Ferroptosis-induced ROS not only exacerbate mitochondrial damage but also further drive imbalances in energy metabolism by activating oxidative stress pathways [36]. For example, ROS accumulation can activate the JNK and p38 MAPK pathways, which promote cellular stress responses and induce programmed death [176,177,178]. At the same time, the dysregulation of energy metabolism creates more favorable conditions for iron accumulation and oxidative damage as well, which creates a vicious circle [92,93,94]. Chen et al. showed that activation of the p38 MAPK pathway affects mitochondrial function and promotes cell death, further reducing the efficiency of ATP synthesis [179]. Experiments by scholars such as Caruso and Jiang have shown that in clinical settings, depressed patients commonly exhibit mitochondrial dysfunction with oxidative damage, which validates the practical implications of this multidimensional mechanism in clinical settings [175,180].

**Figure 4:**
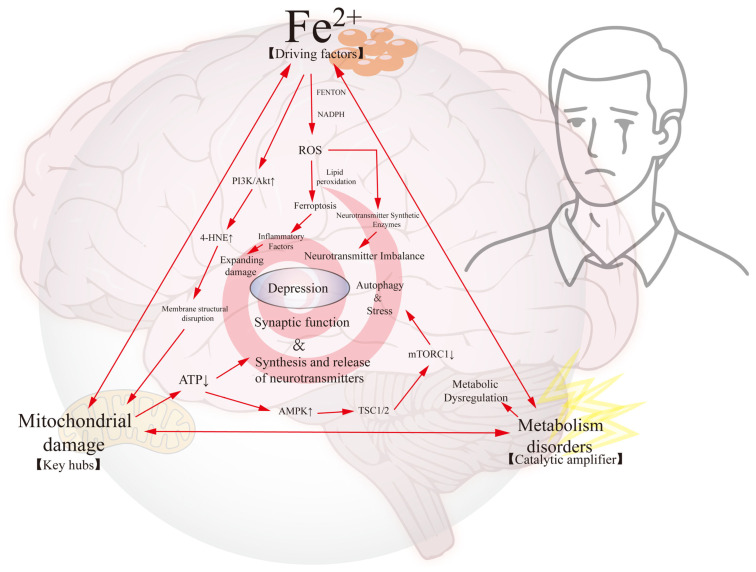
Interactive regulation of ferroptosis, mitochondrial dysfunction, and metabolic disorders in depression. This figure systematically illustrates the interactive regulatory network of ferroptosis, mitochondrial dysfunction, and metabolic disorders in the pathological process of depression. The Fe^2+^-driven Fenton reaction is the driving factor, which is amplified in a cascade through the following core pathways: (1) lipid peroxidation mediated by the ferroptosis pathway triggers membrane structural disruption, which triggers the accumulation of toxic aldehydes, such as 4-HNE; (2) mitochondrial damage hubs are characterized by ATP synthesis impairment and abnormalities in the AMPK/TSC1/2 regulatory axis, leading to energy metabolism remodeling; (3) the mitochondrial dysfunction and metabolic disorders are also affected by the ferroptosis pathway. Key hubs of mitochondrial damage are characterized by ATP synthesis disorders and abnormalities in the AMPK/TSC1/2 regulatory axis, leading to energy metabolism remodeling. Catalytic amplifier exacerbates neurotransmitter synthesis disorders and oxidative stress through mTORC1–autophagy imbalance. These three factors cause synergistic damage through the ROS–inflammatory positive feedback loop, ultimately leading to impaired synaptic plasticity and neurotransmitter homeostasis imbalance, constituting a multidimensional pathophysiological hub for the development of depression.

Taken together, the roles of ferroptosis, mitochondrial damage, and imbalanced energy metabolism in depression constitute an intricate molecular network that drives neuronal decline and mood disorders; the complex interaction network among them is illustrated in Figure 4. From the perspective of molecular mechanisms, the interplay of oxidative stress, mitochondrial damage, and imbalance in energy metabolism triggered by iron ion overload affects the stability of the neural network in the brain, which ultimately leads to the clinical manifestations of depression. Based on the above analysis, we propose a triad of “ferroptosis-mitochondrial fragmentation-metabolic maladaptation”, which together exacerbate the neuropathological process of depression through a complex positive feedback loop, and may become the theoretical basis for molecular typing and precise intervention.

## 5. Outlook

Although we have tentatively recognized the role of ferroptosis, mitochondrial damage, and energy metabolism imbalance in depression, the specific mechanisms need to be further explored. Ferroptosis triggers lipid peroxidation, membrane damage, and neuronal death through excessive accumulation of intracellular iron ions, and key molecules (e.g., GPX4 and FSP1) play an important role in this process; in particular, GPX4 can protect neurons from ferroptosis by reducing lipid peroxides [14,16,55,60,181,182]. Mitochondrial damage leads to disturbed energy metabolism and apoptosis, and the PINK1–Parkin pathway and mitochondrial autophagy play key roles in maintaining the healthy quality of mitochondria [135]. Oxidative stress-induced mitochondrial damage not only disrupts metabolic function but also further exacerbates the pathology of depression by altering synaptic transmission through the Ca^2+^ signaling pathway [121,146]. Therefore, the following key questions remain about its specific mechanisms: (1) The causal relationship between ferroptosis and mitochondrial damage has not been clarified, e.g., whether iron overload directly contributes to electron transport chain dysfunction by inducing lipid peroxidation of mitochondrial membranes (e.g., cardiolipin oxidation) [117]. (2) Metabolic imbalances (e.g., ATP depletion) may modulate ferroptosis sensitivity through the AMPK/mTOR pathway, but the specificity of this mechanism in neurons has not yet been validated [183]. (3) There is a lack of single-cell-level evidence for differences in the response of different brain regions (e.g., hippocampus vs. prefrontal cortex) to the iron–mitochondria–metabolism axis. Future studies need to combine conditional knockout models (e.g., GPX4-flox/flox; Camk2a-Cre) with in vivo calcium imaging to dynamically resolve the spatiotemporal contribution of ferroptosis and mitochondrial damage [184]. It is necessary to explore the interplay between ferroptosis, mitochondrial damage, and metabolic imbalance, with insights into their manifestations in different brain regions and neurons, and how these mechanisms are molecularly regulated to maintain neuronal homeostasis [20].

The limited effectiveness of current single-target treatments in addressing the complexity of depression highlights the potential of multi-target interventions. Existing treatments rely heavily on selective serotonin reuptake inhibitors (SSRIs), which provide rapid symptomatic relief but fail to fundamentally alter the pathomechanisms, and this course of treatment is often accompanied by side effects such as nausea, diarrhea, insomnia, and erectile dysfunction [185]. Therefore, future therapeutic strategies may combine multiple interventions such as iron homeostasis regulation, antioxidant therapy, and mitochondrial protection. For example, iron accumulation can be controlled by inhibiting the iron transporter protein FERROPORTIN or regulating the hepatocyte ferritin HEPCIDIN, and oxidative damage can be minimized by increasing superoxide dismutase (SOD) or catalase (CAT) activity [186,187]. Activation of mitochondrial deacetylase (SIRT3) improves mitochondrial function, etc. [188,189]. In terms of clinical translation, a Master Protocol Trial design, such as the Umbrella Trial, could be used to evaluate the efficacy of hepatocyte ferritin monoclonal antibodies (vamifeport) in parallel with a SIRT3 activator (e.g., nicotinamide riboside, a precursor of NAD+) in patients stratified by different biomarkers (e.g., the plasma 8-OHdG high-level subgroup) in patients with different biomarker stratification [190]. In addition, spatial metabolomics (MALDI-MSI) can localize the distribution characteristics of lipid peroxidation products (e.g., 4-HNE) in the cerebrospinal fluid of patients, providing a basis for individualized treatment [191].

On the other hand, although iron chelators, antioxidants, and mitochondrial protectors have shown potential in animal models, their effectiveness in human clinical applications still needs to be verified [41]. Therefore, we should carry out a large number of clinical trials in the future to evaluate the efficacy, safety, and side effects of these treatments, especially to control the risks that may occur in patients in long-term applications. At the same time, the heterogeneity of depression also requires the development of individualized therapeutic strategies, which can be based on the patient’s genomic and metabolomic information to select the most appropriate treatment options. With the deepening of gene editing technologies (e.g., CRISPR/Cas9) and stem cell research, precise intervention methods for specific pathological subtypes may emerge in the future, such as enhancing neuronal plasticity by improving the expression of the BDNF gene through gene editing or replacing damaged neurons and promoting nerve regeneration through stem cell therapy, as well as constructing patient-specific neuronal models of depression by induced pluripotent stem cells (iPSCs), combined with gene editing (e.g., correction of the BDNF Val66Met mutation) and organoid cultures to mimic the pathology of different brain regions and to screen for targeted drugs, thereby further improving the therapeutic management of depression [192,193].

## 6. Conclusions

The convergence of ferroptosis, mitochondrial damage, and cerebral metabolic remodeling establishes a self-amplifying pathological triad in depression through three interlocked mechanisms: (1) GPX4 inactivation-driven lipid peroxidation disrupts neuronal membrane integrity while impairing mitochondrial respiratory chain complexes (I/III/IV) and releasing DAMPs that activate microglial NLRP3 inflammasomes; (2) mtDNA leakage from fragmented mitochondria perpetuates neuroinflammation via cGAS-STING-TLR9 crosstalk, with compensatory hippocampal glycolytic shifts paradoxically suppressing mitochondrial biogenesis through HIF-1α/BNIP3-mediated mitophagy dysregulation; (3) iron–redox–metabolic feedback loops sustain this triad via hepcidin-mediated ferroportin degradation and aconitase-2 inactivation. On this basis, stratified precision therapeutic strategies (e.g., targeting hepcidin for iron metabolism modulation, combining the mitochondria-targeted antioxidant SS-31 with SIRT3 activators) have demonstrated the potential to break away from monotherapies, and a number of clinical trials have preliminarily validated its safety. Future studies need to combine single-cell spatial genomics, CRISPR screening, and iPSC disease modeling to resolve the heterogeneity of neuronal subtypes in different brain regions in response to the iron–mitochondria–metabolism axis, and to clarify the causality of ferroptosis (point of contention: some studies suggest that it may be a concomitant event of neurodegeneration). By integrating mechanism resolution and technological innovation, depression treatment will move towards an era of multidimensional intervention and biomarker-driven individualization.

## Data Availability

This article is a narrative review synthesizing existing literature on the ferroptosis–mitochondrial axis in depression, with a focus on the feedforward loop linking oxidative stress, metabolic homeostasis dysregulation, and neuroinflammation. No new experimental data were generated or analyzed in this study. All theoretical frameworks, mechanistic hypotheses, and clinical evidence discussed are derived from publicly available sources, including peer-reviewed publications (e.g., PubMed, Web of Science), as cited in the reference list. Readers seeking access to primary data underlying the reviewed studies are directed to the original publications cited in the text. No supplementary datasets, computational code, or experimental materials were created during the preparation of this review.
